# The Swiss Health Insurance Literacy Measure (HILM-CH): Measurement Properties and Cross-Cultural Validation

**DOI:** 10.1186/s12913-022-08986-0

**Published:** 2023-01-26

**Authors:** Tess L. C. Bardy

**Affiliations:** Department of Health Sciences and Medicine & Center for Health, Policy and Economics, University of Lucerne, Frohburgstrasse 3, CH-6002 Lucerne, Switzerland

**Keywords:** Health insurance literacy, Psychometric properties, Multidimensional instrument, Measurement invariance/equivalence, Construct validity, Reliability and validity, Factor analysis

## Abstract

**Background:**

Most consumers face difficulties when choosing and navigating health insurance plans. Health insurance literacy (HIL) has been discussed as a critical lever to ensure efficient choices and navigation in choice-based health insurance systems. Still, existing evidence about HIL mainly comes from the US, and the only validated scale, the Health Insurance Literacy Measure (HILM), may not be adequate to measure HIL outside US samples. This paper describes the measurement properties of the Swiss Health Insurance Literacy Measure (HILM-CH), the first scale to measure HIL in Switzerland.

**Methods:**

The items of the HILM-CH were adapted from the HILM in German, French, and Italian. A panel of experts refined it to ensure its suitability for the Swiss context. The final version of the HILM-CH contains 21 items, and other relevant measures were administered in the Swiss Health Insurance Literacy Survey to a sample of 6036 insurees. Measurement properties were investigated overall and per linguistic group. Internal reliability was determined using Cronbach’s alphas. Criterion validity was examined through convergent and concurrent validity of the HILM-CH. The construct validity was assessed using factor analysis. Measurement invariance of the HILM-CH between linguistic regions was further evaluated using multiple-group confirmatory factor analyses.

**Results:**

The HILM-CH had acceptable to good reliability (alphas between 0.70 and 0.91). Concurrent and convergent validity showed that HILM-CH is a good measurement of HIL. Factor analysis revealed a four-factor model and showed an acceptable fit to the data (CFI= 0.977; TLI = 0.974; RMSEA = 0.061; SRMR = 0.032). Using the established four-factor model, measurement invariance was established across Switzerland’s German, French, and Italian-speaking regions.

**Conclusions:**

The HILM-CH is a reliable and valid measure of HIL across Switzerland’s German, French, and Italian-speaking regions. It can be used in future research to find associations between HIL and individual characteristics.

**Supplementary Information:**

The online version contains supplementary material available at 10.1186/s12913-022-08986-0.

## Introduction

Switzerland has a choice-based health insurance system for basic mandatory coverage allowing consumers various choices of health insurance plans [1]. The system assumes that consumers can and do make informed choices about the plan they enroll in [2–4]. Informed choices require knowledge of cost-sharing features such as copayments, deductibles, out-of-pocket expenditures, and the specific services covered by the plan. Once enrolled, consumers must also understand the type of physician network and the consequences of going outside the network, the role of primary care providers, or how to obtain specific care referrals [2, 5]. Consumers may further distinguish between an insurer’s reputation, such as the ease of processing claims when choosing a health insurance plan [6].

Previous research has shown that most consumers lack a basic health insurance understanding. Failure to understand health insurance prevents consumers from being proficient in finding, choosing, and using health insurance plans [4]. This results in inefficient plan selection, that is to say, a plan that does not meet consumers’ health and financial needs, which, in turn, creates overspending and delayed (if not forgone) care [7]. Furthermore, even if the plan meets their needs, a lack of health insurance knowledge might lead to the inefficient use of the plan, eventually facing similar consequences. For instance, enrollees might experience greater barriers to care, such as delays in treatments, if they fail to understand the restrictions regarding physician choice and access included in the plan [2].

Health insurance literacy (HIL) has been discussed as a critical lever to ensure efficient choices and navigation in choice-based health insurance systems [5]. HIL is defined as “the degree to which individuals have the knowledge, ability, and confidence to find and evaluate information about health plans, select the best plan for their own (or their family’s) financial and health circumstances, and use the plan once enrolled. [7]” Thus, the concept of HIL may explain the barriers, such as the incapacity to understand the health and financial implications of health insurance plans, that prevent consumers from making efficient plan choices and use [5, 8, 9]. For instance, HIL measures revealed associations between consumers’ ability to choose and navigate health insurance plans and their level of education, age, financial and marital status, and the healthcare experience [10–12]. Paez and Mallery [13] showed that low-educated, young individuals, migrants, or people who do not often deal with the health care system have the lowest HIL and are more likely to forgo care due to costs. Another recent study underlined that low HIL levels are associated with lower confidence in using plans to access care [14].

Still, existing evidence about HIL mainly comes from the US [15], and the most recent and only validated scale to measure HIL, the Health Insurance Literacy Measure (HILM), may not be adequate to measure HIL outside the US samples. Developed by Paez et al. [11] for the US private health insurance market, the HILM is a multidimensional instrument that measures self-perceived (and system-related) issues and barriers in selecting, understanding and using health insurance plans. A recent systematic literature review revealed the potential of the HILM for cultural adaptation [15].

Switzerland depicts a unique opportunity to adapt the HILM as previous research evidenced consumers’ difficulties understanding and navigating health plans. For instance, 40% of the population reported having trouble finding information about health plans [16], and 17% did not know their health plan details [17]. This comes as a major concern since 22% of the Swiss population faced unmet health care needs due to financial burden [18], together with the highest share of out-of-pocket health care spending (5.8%) among the OECD members [19].

Therefore, this paper proposes to examine the properties of the Swiss Health Insurance Literacy Measure (HILM-CH), an adaptation of the HILM to fit the Swiss context developed specifically for that study. First, the paper investigates the measurement properties of the HILM-CH, i.e., its validity and reliability. Second, and because Switzerland is characterized by three main linguistic regions (German, French, and Italian), the paper examines the measurement invariance of HILM-CH across the three linguistic groups that are culturally different [20]. The created measure would allow future research to understand HIL levels and better understand the self-perceived barriers to choosing and navigating Switzerland's health plans. Further, while it would allow for international comparisons, a tool validated in different languages could be a first step in adapting the instrument for surrounding countries, such as Germany, France, Austria, or Italy, that have choice-based health insurance systems for supplementary health insurance.

## Methods

### Data collection and participants

Between September and October 2021, 6036 online interviews with an average duration of 15 minutes were conducted in German, French, and Italian to collect data for the Swiss Health Insurance Literacy Survey. Data collection was conducted by intervista AG, a private market research company that operates under the General Data Protection Law and the Federal Act on Data Protection in Switzerland. Participants were Swiss residents aged between 26 and 75 years and were selected based on predefined quotas for gender, age, education, and language region to ensure the representativeness of our sample. Corrections for over- and underrepresentation of specific subpopulation groups in the online panel were applied using sample weights. All analyses  were run on R version 4.1.2 [21].

### Survey instrument

Established by Paez et al. [11], the HILM is composed of 21 survey items, each of which is intended to address one of four dimensions (or scales) of HIL:

1. Selecting health insurance
  a Scale 1: Confidence in choosing health insurance
  b Scale 2: Comparing health plans
2. Using health insurance
  a Scale 3: Confidence in using health insurance
  b Scale 4: Proactive use of insurance

Each of the 21 items evaluates self-reported health insurance literacy on a four-point Likert scale from 1 “not confident/likely at all” to 4 “very confident/likely.” Scores can be averaged from the single items to build a scale score, overall and for each HIL domain. Higher scores indicate higher self-reported HIL.

Items were drawn from the HILM to create the HILM-CH. Before starting with the fieldwork, the HILM-CH was tested and validated by different experts from the Swiss health system to ensure the suitability of all the items for the Swiss context. For instance, one additional item was included in scale 2: “when comparing health plans, how likely are you that you find out what are the differences between them?” to understand the general barriers consumers face when choosing health plans. One item in scale 3, “how confident are you that you know most of the things you need to know about using health insurance?” (scale 3), was dropped to avoid redundancy. Further, any reference to the employer providing health insurance was dropped as in Switzerland employers do not provide health insurance plans. As the HILM, the final version of the HILM-CH comprised 21 items.

The HILM-CH was then translated from English to German, French, and Italian following Epstein et al. [22]. Two translations were done for each language by native speakers. Both versions were compared to compose a draft questionnaire in each of the three languages. The HILM-CH was administered in German, French, and Italian. The three versions are available upon request. The English version is available in the additional files (see Additional file 1). An online pilot study (N=184) ensured the understandability of the HILM-CH in the three languages. No changes to the instrument were necessary, and the HILM-CH was included in the Swiss Health insurance literacy survey with additional questions about socio-demographic background, health status, and current health insurance choices. Data from the pilot study were excluded from the final dataset.

### Statistical analysis

#### Measurement properties

The overall and per language reliability was examined in the form of internal consistency to reveal the ability of HILM-CH to have interrelated items. It assumes that multiple items measure the same underlying latent construct. In the case of the HILM-CH, items can be allocated into four different scales, but when combined, measure overall self-perceived HIL. A high Cronbach’s alpha indicates that items are highly correlated and, therefore, that the instrument is reliable, with alpha ≥ 0.70 being acceptable [23].

The validity of an instrument refers to its ability to measure what it is supposed to measure [24]. First, and following Paez et al. [11], the criterion validity of the HILM-CH was examined to assess if participants’ scores are correlated with other variables that are suspected to be correlated with HIL. As part of the criterion validity, concurrent and convergent validity were investigated. Concurrent validity refers to the correlation of the instrument with any variables that should be correlated with the latent construct being measured (i.e., HIL). In contrast, convergent validity refers to the correlation between the instrument and another measure measuring the same construct [25]. Both concurrent and convergent validity were assessed using Spearman’s rank correlation coefficients (rho), where rho < 0.25 was small, rho ≤ 0.5 moderate, rho ≤ 0.75 good, and rho > 0.75 excellent [26].

Concurrent and convergent validity were derived using information drawn from the data. Concurrent validity was investigated by comparing the HILM-CH scores with a subjective statement integrated into the survey as a standard: “Would you say that your knowledge about the Swiss health insurance system is very good, good, acceptable, mediocre, or not good at all?”

Convergent validity was derived by comparing the instrument’s scores with the scores obtained on an objective set of multiple-choice (true or false) questions about the Swiss health insurance system implemented in the survey. Choices were provided with “I don’t know” as an additional answer option to reduce respondents' likelihood of guessing when they are not sure about the answer. Scores for the objective questions were determined by summing the number of correct responses. In both the concurrent and convergent validity, a strong positive association would indicate that the health insurance literacy scales are linked to higher health insurance knowledge and ability, confirming the instrument’s validity.

Second, the construct validity of the HILM-CH was investigated using factor analysis to examine whether the instrument purports to measure the underlying construct. The appropriateness of factor analysis was evaluated using the Kaiser-Meyer-Olkin measure of sample adequacy (KMO) and Bartlett’s test of sphericity. Patterns of variance and correlation among answers to the different items representing each dimension of HIL were investigated using exploratory factor analysis (EFA). Results of the EFA were then tested using confirmatory factor analysis (CFA).

In the EFA, the various items of the instrument were examined to gather them into manageable sets of underlying concepts [27]. The relationship between the latent construct (i.e., HIL) and the observed answers to the items that compose the HILM-CH are modeled using factor loading coefficients. Ideally, each item loads (i.e., correlates) strongly on a single factor (i.e., a factor loading ≥ 0.3 [28]). Weighted least square factoring method was used due to the ordinality of the data. The number of factors to extract was based on a scree plot and parallel analysis, and the rotated factors’ conceptual meaningfulness using Promax rotations [29]. Given the factor pattern, names for the factors were defined based on the content of the items that strongly loaded upon them.

While EFA aims to identify the factor structure present in a set of items, CFA tests the hypothesized factor structure proposed by the former. It investigates the correlation among variables to see if they are consistent with the factor structure. Different indicators were referred to assess the fit in the extracted factor model, including the comparative fir index (CFI) and the Tucker-Lewis index (TLI), the root mean squared error of approximation (RMSEA), and the standardized root mean squared residual (SRMR), with CFI > 0.9, TLI > 0.9 depicting a good incremental fit, and RMSEA ≤ 0.08, SRMR ≤ 0.08 depicting an acceptable absolute fit [30]. Chi-squared test (${\upchi }^{2}$) and its corresponding degrees of freedom (df) were reported for misspecification.

#### Measurement invariance

Finally, since Switzerland is a multi-cultural country with three main linguistic regions, cross-cultural validity of our instrument was investigated. If invariance is observed, the HIL construct can be measured across the different Swiss cultural groups, and it is possible to argue in favor of the cross-cultural validity of the HILM-CH [31].

To test the invariance of the HILM-CH across German, French, and Italian-speaking participants, multiple group confirmatory factor analyses (MGCFAs) were run. Given the ordinal nature of the data, weighted least squares mean and variance adjusted (WLSMV) estimation were run. For purposes of model identification, latent variables were standardized. Configural, metric, and scalar invariance were tested in nested MGCFA models. Additionally, the level of invariance was tested by reporting the difference in fit indices between that of the more constrained model to that of the next less constrained model [32]. Configural invariance means that factor patterns do not vary across groups. Metric invariance states that identical items are loading on identical factors across groups and that the loading magnitudes are the same across groups for each item. Finally, scalar invariance imposes configural and metric invariance constraints and adds that intercepts are the same across groups [32]. An achievement of measurement invariance would indicate that HILM-CH is a cross-culturally valid instrument, measuring the same underlying HIL construct across the three main Swiss linguistic regions. For assessment of model fit, different fit indices were reported: CFI, RMSEA, and SRMR, and their respective changes when increasing model constraints. Following Chen [33], thresholds for achieving loading invariance are variations ≥ -0.010 for CFI and ≥ 0.015 for the RMSEA or ≥ .030 for SRMR. To have scalar invariance, changes in thresholds ≥ -0.010 for CFI and ≥ 0.015 for RMSEA or ≥ 0.016 for SRMR were accounted for. Chi-squared statistics were not reported due to their sensitivity to the sample size [34].

## Results

### Sample description

Socio-demographics, health characteristics, and health insurance choices for the total sample and each linguistic region are presented in Table 1. Of the 6036 respondents, 70% were German-speaking, 23% French-speaking, and 6% Italian-speaking. In comparison, in 2019, 62% of the total Swiss population were German-speaking, 23% French, and 8% Italian [20]. All respondents are Swiss residents and most of them had Swiss nationality (91.2%). Sixty-four per cent completed primary or secondary education. In comparison, they were 56% in 2020 [35]. On average, respondents were 49.6 years old (SD = 13.74). They reported an average of 3.9 doctor visits in the last 12 months (SD = 5.31), and the majority was not suffering from any chronic conditions. Regarding health insurance choices, most of the sample chose a yearly deductible of 300 or 2500 Swiss Francs; 55.6% had a family doctor health plan and low out-of-pocket spending. Language differences characteristics are reported in the last column using Fisher’s exact test and iterative proportional fitting.

**Table 1:** Sample characteristics

	**Total**		**German**		**French**		**Italian**		
	***N***	**%**	***N***	**%**	***N***	**%**	***N***	**%**	***P***
**Total**	6036	100	4274	70.81	1409	23.33	353	5.85	
**Age (mean, SD)**	49.62	13.74	50.08	13.80	48.87	13.69	46.98	12.75	0.037
**Gender**									0.118
Male	3016	49.97	2126	49.7	694	49.3	195	55.3	
Female	3020	50.03	2148	50.3	714	50.7	158	44.7	
**Swiss nationality**^a^									<0.01*
Yes	5503	91.17	3995	93.5	1257	89.2	251	71.1	
No	533	8.83	279	6.5	152	10.8	102	28.9	
**Education**^b^									<0.01*
Primary/Secondary	3846	63.72	2562	65.63	585	59.00	404	59.54	
Tertiary	2190	36.28	1341	34.37	597	41.00	274	40.46	
**Doctor visits** **last 12 months (mean, SD)**	3.92	5.31	3.95	5.26	3.87	5.41	3.77	5.51	0.597
**Chronic conditions**									<0.01*
Yes	2307	38.2	1669	39.0	536	38.1	102	29.0	
No	3607	59.8	2527	59.1	839	59.6	240	67.9	
Don’t know	122	2.0	78	1.8	33	2.3	11	3.1	
**Yearly deductible**									<0.01*
300 CHF	2331	38.6	1665	71.4	535	23.0	131	5.6	
500 CHF	601	10.1	374	62.2	195	32.4	32	5.3	
1 000 CHF	221	3.7	173	78.3	38	17.2	10	4.5	
1 500 CHF	390	6.5	277	71	97	24.9	16	4.1	
2 000 CHF	217	3.6	170	78.3	39	18	8	3.7	
2 500 CHF	2159	35.8	1523	70.6	484	22.4	152	7.0	
Don’t know	117	1.9	92	2.2	20	1.4	5	1.4	
**Type of health plan**^**c**^									<0.01*
Basic	1114	18.4	723	16.9	312	22.2	78	22.1	
HMO	516	8.5	455	10.6	42	3.0	19	5.3	
Telemedicine	959	15.9	747	17.5	163	11.	49	13.9	
Family doctor	3174	52.6	2204	51.6	795	56.5	174	49.2	
Other	107	1.8	64	1.5	34	2.5	9	2.5	
Don't know	167	2.8	81	1.9	62	4.4	25	7.0	
**Out-of-pocket expenditures**									0.239
None	609	10.1	444	10.4	125	8.9	40	11.4	
< 300 CHF	1543	25.6	1106	25.9	337	23.9	99	28.2	
300 - 499 CHF	1184	19.6	817	19.1	302	21.5	65	18.4	
500 - 999 CHF	1215	20.1	846	19.8	307	21.8	62	17.6	
1000 - 1499 CHF	602	10.0	421	9.9	144	10.2	36	10.3	
1500 - 2499 CHF	296	4.9	221	5.2	55	3.9	20	5.6	
≥ 2500 CHF	264	4.4	192	4.5	59	4.2	13	3.7	
Don’t know	323	5.4	227	5.3	79	5.6	17	4.9	

Note: Reported numbers depict the weighted characteristics of the total sample and per linguistic region (German, French and Italian).

^a^Individuals holding the Swiss nationality hold it from birth or obtained it after ten years of residency. Individuals who do not have the Swiss nationality do not have the Swiss passport but an official document proving that they are residents.

^b^Tertiary education refers to respondents who obtained a degree from a University of Applied Sciences (“Höhere Fach- oder Berufsausbildung” and “Fachhochschule”), a college of education (“Pädagogische Hochschule”), a Swiss University, or a Swiss Federal Institute of Technology (“Eidgenössische Technische Hochschule”). All other (un)completed education levels were assigned to Primary and Secondary education.

^c^Type of health plans: “basic” refers to a plan with a free choice of practitioner. “HMO” (health managed organization), “Telemedicine,” “family doctor,” and “other” refer to a type of plan where the first point of contact (gatekeeper) is different and specific. Individuals who do not comply with the first point of the contact face extra costs.

^*^P<0.01

Source: Swiss Health Insurance Literacy Survey 2021.

### HILM-CH score description

The mean and standard deviation of the scores of the four subscales of the HILM-CH are shown in Table 2. Internal consistency estimates are shown in the same table and will be discussed in the next section. Respondents self-rated their HIL level as follow: scores range between 2.74 (SD = 0.22) and 3.00 (SD = 0.16). Overall, French-speaking respondents displayed lower scores per scale than German- and Italian-speaking respondents. For instance, their average confidence when using health insurance (Scale 3) was equal to 2.65 (SD = 0.64), while it was 2.75 (SD = 0.68) for German-speakers and 2.68 (SD = 0.66) for Italian-speakers. The results also suggest that it is easier for respondents to be more proactive when they already have a health plan and seek information/help than when they have to navigate among the plans to select one (as suggested by Paez et al. [11]). Finally, ANOVA post hoc weighted pairwise comparisons using Tukey’s honestly significant difference (HSD) depicted significant cultural differences between the German and Latin regions. Additional files provide more information about the distribution of the scores and differences across groups (see Additional Table 1 and Additional Table 2).

**Table 2:** Means, Standard Deviations, and Cronbach's alpha values for the HILM-CH

	**Total (*N*=6036)**			**German (*N*=4274)**				**French (*N*=1409)**			**Italian (*N*=353)**		
	**Mean**	**SD**	**α**	**Mean**^**a**^		**SD**	**α**	**Mean**^**a;b**^	**SD**	**α**	**Mean**^**a;b**^	**SD**	**α**
**Scale1** Confidence choosing	2.86	0.16	0.83	2.88		0.56	0.82	2.83	0.56	0.85	2.82	0.57	0.83
**Scale2** Comparing plans	2.82	0.10	0.91	2.87		0.63	0.91	2.69	0.63	0.91	2.81	0.63	0.91
**Scale3** Confidence using	2.74	0.22	0.86	2.79		0.68	0.86	2.65	0.64	0.85	2.68	0.66	0.85
**Scale4** Being proactive	3.00	0.16	0.75	3.05		0.63	0.75	2.90	0.62	0.77	2.92	0.60	0.70

Note: α, Cronbach’s alpha values. Weighted values are reported.

^a^Tukey’s HSD ANOVA post hoc pairwise comparison test depicted statistical difference (p<0.001) between German and French and German and Italian for each scale score.

^b^Tukey’s HSD ANOVA post hoc pairwise comparison test depicted no statistical difference between French and Italian for each of the four scale scores.

Source: Swiss Health Insurance Literacy Survey 2021.

### Measurement properties

#### Reliability

Table 2 depicts the Cronbach’s alphas per scale for the total sample and the three linguistic regions. Scale 1 has an alpha ranging from 0.82 to 0.85, scale 2 of 0.91, scale 3 from 0.85 to 0.86, and scale 4 from 0.70 to 0.75. These values indicate the acceptable to good reliability of the HILM-CH.

#### Validity

Table 3 shows the association between the scores of the four scales of the HILM-CH and the self-reported statement on health insurance knowledge (concurrent validity), as well as with the score obtained for the objective set of questions on the Swiss health insurance system (convergent validity).

**Table 3:** Concurrent and convergent validity - Spearman's rank coefficient correlations

	**Concurrent validity**				**Convergent validity**			
**Scores**	**Total (*N*=6036)**	**German** **(*N*=4274)**	**French** **(*N*=1409)**	**Italian** **(*N*=353)**	**Total (*N*=6036)**	**German** **(*N*=4274)**	**French** **(*N*=1409)**	**Italian** **(*N*=353)**
**Scale1** Confidence choosing	0.58	0.59	0.57	0.57	0.17	0.16	0.18	0.22
**Scale2** Comparing plans	0.43	0.44	0.42	0.46	0.15	0.14	0.14	0.26
**Scale3** Confidence using	0.44	0.44	0.46	0.47	0.11	0.10	0.12	0.18
**Scale4** Being proactive	0.30	0.30	0.32	0.36	0.11	0.11	0.10	0.17

Note: rho correlation coefficients < 0.25 are considered as small; ≤ 0.5 as moderate; ≤ 0.75 as good; > 0.75 as excellent.

All coefficients are significant at 0.001. Weighted values are reported.

Source: Swiss Health Insurance Literacy Survey.

Moderate to good concurrent validity was found, with correlation coefficients ranging from 0.30 to 0.58 for the full sample and per language group. The self-assessed measure was positively correlated with the objective pool of items. The comparison was higher for the selection scales “confidence choosing” and “comparing health plans” with correlations of 0.17 and 0.15, respectively. The correlation was 0.11 for both “confident using” and “being proactive”. In line with Paez et al. [11], this result suggests that consumers tend to overestimate their HIL. It is also a sign that a higher endorsement of confidence and self-assessed behavior regarding choosing and using health insurance is likely related to true health insurance knowledge and skills.

**Fig. 1:**
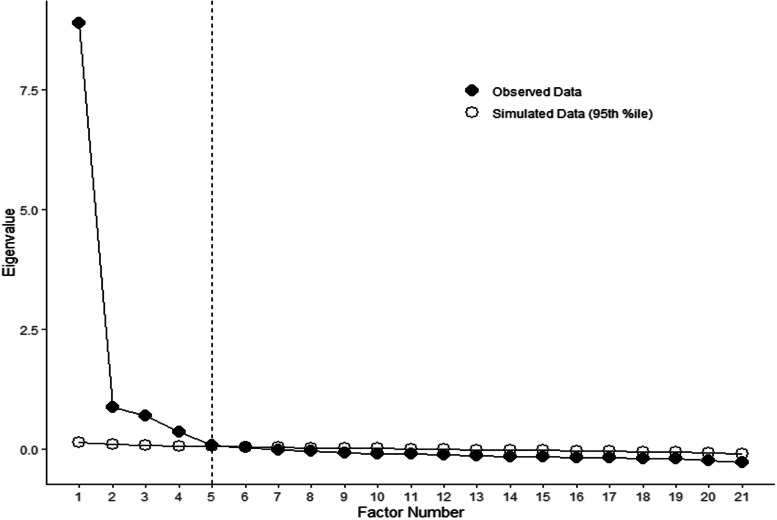
Scree plot and parallel analysis for exploratory factor analysis

Regarding the construct validity of the HILM-CH, both KMO and Bartlett’s test for sphericity measures indicated that the data were appropriate for factor analysis (KMO = 0.96 and ${\upchi }^{2}$= 66519.85, df = 210, *p* < 0.001 [36, 37]). A scree plot of the eigenvalues evidenced a departure from linearity coinciding with a four-factor solution [38] (see Fig. 1). In line with the expectations that the HILM-CH reflects four dimensions of HIL, the fifth factor was not retained as suggested by the parallel analysis due to difficulty in characterizing it and poor factor loadings [39]. Thus, a 4-factor model was evaluated wherein each item could load onto all factors [28].

Table 4 summarizes the results of the EFA. Correlation among factors after CFA is reported in this table and will be discussed afterward. Using a cutoff value of 0.4, each item loaded on a single factor. No cross-loading was reported, indicating that each variable represents the factor on which it loaded [40]. Except for items 2 and 5, uniqueness was lower than 0.6, stressing that the four factors explain the factor model well [41]. To check for model fit improvements, the CFA was run without items 2 and 5 and did not show significant improvement in the model fit (CFI = 0.979, TLI= 0.975, RMSEA = 0.064, SRMR = 0.031), thus the initial model was kept.

**Table 4:** EFA 4-factor solution and correlation among factors of the HILM-CH after CFA

**Item**	**HILM-CH**	**Mean**	**SD**	**Factor 1**	**Factor 2**	**Factor 3**	**Factor 4**	**Uniqueness**
**Scale 1: confidence in choosing health plans – How confident are you that you..**.								
1	…understand the concepts and terms about health insurance plans?	2.80	0.72	.	0.70	.	.	0.47
2	…know where to find the information you need to choose a health insurance plan?	2.88	0.75	.	0.41	.	.	0.73
3	…can estimate what you will have to pay for your care in the coming year (without emergencies)?	2.90	0.73	.	0.88	.	.	0.33
4	…know where to go for financial help if you cannot pay your health insurance plan?	3.04	0.72	.	0.77	.	.	0.42
5	…know which questions to ask in order to choose the health insurance plan that is right for you?	2.57	0.96	.	0.39	.	.	0.72
6	…are choosing a health insurance plan that suits you?	3.00	0.71	.	0.83	.	.	0.42
**Scale 2: comparing health plans – When comparing health insurance plans, how likely it is that you…**								
7	… can find out if you have to pay for certain care yourself?	2.93	0.75	0.75	.	.	.	0.38
8	…find out which doctors and hospitals are covered by an insurance policy?	2.65	0.83	0.79	.	.	.	0.38
9	…can find out how much you have to pay for medicines on prescription?	2.77	0.81	0.80	.	.	.	0.37
10	…can find out how much you have to pay for a visit to the emergency room?	2.80	0.81	0.74	.	.	.	0.43
11	…can find out how much you have to pay yourself for a visit to a medical specialist?	2.95	0.77	0.68	.	.	.	0.43
12	…can find out if an insurance policy covers unexpected costs, such as a hospitalization?	2.80	0.77	0.56	.	.	.	0.45
13	…can find out what the differences are between insurance plans?	2.84	0.77	0.78	.	.	.	0.38
**Scale 3: confidence in using health plans – How confident are you that you…**								
14	…can find out how much of the costs your health insurance company will reimburse?	2.44	0.89	.	.	.	0.56	0.51
15	…can find out how much you have to pay out of your own pocket?	2.80	0.78	.	.	.	0.82	0.28
16	…know which questions to ask your health insurance company if you have a problem with a reimbursement?	2.82	0.77	.	.	.	0.84	0.27
17	…know what to do if your health insurance company refuses to pay for care that you think you should be reimbursed?	2.93	0.80	.	.	.	0.55	0.46
**Scale 4: proactive use of insurance – When using your health insurance plan, how likely is it that you…**								
18	…will find out what is and is not covered by your health insurance plan before you receive a certain care?	2.94	0.79	.	.	0.63	.	0.40
19	…will contact customer service to ask what care is covered by your health insurance plan?	3.17	0.78	.	.	0.75	.	0.59
20	…can find out whether a doctor has a contract with your health insurance company before you visit a doctor?	2.83	0.92	.	.	0.55	.	0.59
21	…look at the overviews of your health insurance plan to see what you still have to pay and what the health insurance company has reimbursed?	3.09	0.81	.	.	0.55	.	0.59
**Correlation among factors after CFA**								
	**Factor 1** Comparing plans			1.00				
	**Factor 2** Confidence choosing			0.69*	1.00			
	**Factor 3** Being proactive			0.75*	0.72*	1.00		
	**Factor 4** Confidence using			0.60*	0.53*	0.65*	1.00	

Note: Blank cells represented by a “.” indicate that the absolute factor loadings were lower than 0.39. Promax rotation was applied to the factor loadings. Weighted values are reported.

^*^: all rho correlation coefficients are significant at < 0.001.

Source: Swiss Health Insurance Literacy Survey 2021.

Following Paez et al. [11] and consistent with the individual items, factors 1 and 2 were labeled after the domain on choosing health insurance, “comparing plans” and “confidence in choosing,” respectively. Factors 3 and 4 were labeled after the domain on using health insurance “being proactive” and “confidence in using,” respectively.

As part of the CFA to test the four-factor model, the fit indices confirmed the model (CFI= 0.98; TLI = 0.97; RMSEA = 0.06; SRMR = 0.03). Correlation coefficients among the four factors are reported in Table 4. Factors’ correlations range from rho = 0.60 between “confidence using” and “confidence choosing” to rho = 0.75 for “comparing plans” and “being proactive”, depicting a good positive relationship among the factors. Further information about the correlation of the four factors is available in Additional files (see Additional file 2) [42].

### Measurement invariance

Fit indices for the MGCFAs are reported in Table 5. The three baseline models were estimated separately for the three linguistic regions (German, French, and Italian) and fit well and MGCFAs nested models were run to test for configural, metric, and scalar invariance across the three language groups.

**Table 5:** Measurement invariance of HILM-CH across the three main Swiss linguistic regions

**Model**	**Fit indices**^**a**^						**Model comparison**	$\Delta$**CFI**	$\Delta$**RMSEA**	$\Delta$**SRMR**
	χ^2^	**df**	**P value**	**CFI**	**RMSEA**	**SRMR**				
**Baseline**										
All	4319.33	183	< 0.001	0.977	0.061	0.032				
German	2736.84	183	< 0.001	0.979	0.060	0.032				
French	1509.93	183	< 0.001	0.969	0.071	0.037				
Italian	619.23	183	< 0.001	0.977	0.059	0.070				
**Model 1** Configural	4959.33	549	< 0.001	0.976	0.063	0.038				
**Model 2** Metric	3683.35	583	< 0.001	0.983	0.051	0.037	2 vs. 1	0.007	-0.012	-0.001
**Model 3** Scalar	5559.53	659	< 0.001	0.974	0.061	0.034	3 vs. 2	-0.009	0.01	-0.003

Note: *HILM-CH* Swiss Health Insurance Literacy Measure,$\chi^2$ chi-square values, *df* = degree of freedom, *CFI* comparative fit index, *RMSEA* root mean square error of approximation, *SRMR* standardized root mean square residuals. Change in fit indices is represented by$\Delta$. *N*=6036.

^a^ Robust values.

Source: Swiss Health Insurance Literacy Survey 2021.

Configural invariance was confirmed (model 1) with a good fit of the data when the factor pattern was equal across groups. The fit was very good when constraining the factor loadings to be equal across groups (model 2). Compared with model 1, the fit of model 2 was better than that of the configural model, as evidenced by the increase in CFI and the decrease in RMSEA and SRMR. Thus, metric invariance across the three linguistic groups could be demonstrated. In model 3, where the equal intercepts constraint was added, the fit was still good with a change in the CFI fit ≤ 0.010, RMSEA ≤ -0.015, and SRMR ≤ -0.016, confirming scalar invariance. Therefore, HIL can be measured across the different Swiss cultural groups with the HILM-CH, ensuring cross-cultural validation.

## Discussion

The present study informed the properties and measurement invariance of the HILM-CH for measuring self-perceived HIL among the Swiss population and linguistic groups.

First, reliability of the HILM-CH was demonstrated with an internal consistency underlining an acceptable to good reliability, overall and across the three linguistic groups. The internal consistency was slightly lower in the “being proactive” scale, probably due to the smaller number of items and their lower relevance due to an overall higher uniqueness [43].

Second, the criterion validity of the HILM-CH was investigated by correlating it with additional information from the survey data. Concurrent validity was examined using a subjective statement about respondents’ perception of their knowledge of the Swiss health insurance system. Convergent validity was analyzed using the scores to an objective set of questions about the Swiss health insurance system. Both showed a positive correlation with the instrument scores, indicating that HILM-CH measures what it is intended to measure: HIL [24, 25]. The implications of the concurrent validity results are worth noticing: the correlation between the HILM-CH scores and the answers to the subjective statement about respondents’ general perception of their knowledge of the Swiss health insurance system revealed a strong and positive correlation (between 30% to 60%). Examining multidimensional instruments requires time and comes with other constraints. Ideally, the construct of interest could be captured using a smaller set of questions. In our case, the concurrent validity revealed that using a single subjective statement in a survey could capture part of HIL very well. Although less precise than the 21 items, this comes in handy under financial or time restrictions to assess HIL in the population.

Third, the construct validity assessment using EFA revealed a four-factor structure. The four-factor model solution showed how well HILM-CH could measure HIL through “confidence in choosing,” “comparing plans,” “confidence in using,” and “being proactive” dimensions of HIL as defined by Paez and colleagues [11]. Indeed, each factor loaded highly on a single factor, showing that each item of the HILM-CH participates in explaining HIL [43]. The CFA fit indices underlined an acceptable absolute model fit and a good incremental fit.

Finally, the last part of the present study used MGCFAs to examine HILM-CH measurement invariance. Variations across German-, French-and Italian-speaking respondents were investigated based on the four-factor model established in the factor analysis. Although cultural differences exist between the German- and Latin-speaking regions, configural, metric, and scalar invariance were demonstrated across the three groups. Thus, the measure maintained measurement equivalence/invariance across groups, suggesting that the HILM-CH measures a meaningful construct across diverse cultures and languages [44].

### Limitations

The current findings should be interpreted with consideration of some limitations. First, the survey ran between September and October 2021. This is one month before consumers should notify their insurers about their willingness to switch their health plans. At that time, brokers at least partly start reaching out to insurees to inform them about possible changes in health insurance premiums and plans. Various online advertisements have also started becoming available. While more information does not increase people’s HIL [45], the pandemic of Covid-19 might have triggered the willingness of Swiss people to be more informed about the health insurance system (e.g., due to job loss and increase in financial burdens). It is worth noticing that more than 46% of the respondents were infected by the coronavirus or knew someone who had been infected at the time of the survey.

Second, although the HILM-CH was validated and cross-validated it in German, French, and Italian, this does not include a comparison with the English American the original version. Further examinations of the measurement equivalence between the HILM and HILM-CH should be carried out to compare associations between HIL and individuals characteristics in future research.

Third, although the four-factor model was validated, the scale “being proactive” showed lower scores and factor loadings than the other scales. A deeper analysis of the fourth scale would benefit the HILM-CH.

## Conclusion

While literature showed that consumers have difficulties choosing and navigating health insurance [10–12, 14], little evidence about the mechanisms that impede people from making efficient plan selection and navigation is still not understood. HIL has been emphasized in the literature as a way to explain these barriers [7], therefore stressing the need to have more valid and reliable measurement tools outside US samples [15].

The establishment of the validity, reliability, and measurement invariance of the HILM-CH, with minor adaptations only compared to the HILM developed initially in the context of and with a view toward the US health insurance marketplaces, opens the door for researchers to examine the challenges of choice-based health insurance systems more broadly other contexts. In the US, the evidenced associations between HIL levels and individuals’ characteristics lead to the creation of tailored programs, such as workshops or web-based tools [46–49]. These programs enhanced consumers’ navigation in health insurance and improved their HIL. They had higher confidence in choosing and using health plans than consumers who did not have access to these programs [50]. Consequently, they were less likely to delay or forego care due to financial burdens [51].

The HILM-CH provides a promising measurement tool with four domains to measure HIL in the Swiss population and across different groups, characterized by the three linguistic regions. Making available a reliable and valid measurement tool for HIL opens future research to measure HIL in the Swiss population and identify the potential associations with consumers’ characteristics and their ability to choose and navigate the Swiss health insurance system. It hopefully inspires similar research for other countries with choice-based health insurance systems and strengthens the common grounds around HIL.

## Supplementary Information

**Additional file 1.** 

**Additional file 2.**

**Additional file 3.**

**Additional file 4.**

## Data Availability

The data that support the findings of this study are available free of charge upon request after signing a data contract with the Center for Health, Policy and Economics (CHPE) at the University of Lucerne, Switzerland. Contact by email via chpe@unilu.ch with a brief description of the planned research and dissemination of results. Restrictions apply to the availability of data that are part of a broader study and provided by intervista AG. Data users may gain access to datasets only after accepting an agreement to use and cite the data in a proper fashion, for scientific research and education within an academic framework, and following typical scientific, ethical norms of conduct. However, all datasets will be available from corresponding author upon reasonable request.

## Abbreviations

CFA: Confirmatory factor analysis
CFI: Comparative fit index
Df: Degree of freedom
EFA: Exploratory factor analysis
HIL: Health insurance literacy
HILM: Health insurance literacy measure
HILM-CH: Swiss health insurance literacy measure
HSD: Honestly significant difference
KMO: Kaiser-Meyer-Olkin measure of sample adequacy
MGCFA: Multiple groups confirmatory factor analysis
OECD: Organisation for Economic Co-operation and Development
RMSEA: Root mean square error of approximation
SRMR: Standardized root mean square residual
TLI: Tucker-Lewis index
US: United States
WLSMV: Weighted least squares mean and variance adjusted

## References

[1] Daily-Amir D, Albrecher H, Bladt M, Wagner J (2019). On Market Share Drivers in the Swiss Mandatory Health Insurance Sector. Risks..

[2] Cunningham PJ, Denk C, Sinclair M (2001). Do Consumers Know How Their Health Plan Works?. Health Aff (Millwood)..

[3] Kunreuther HC, Pauly MV (2013). McMorrow S.

[4] Bhargava S, Loewenstein G (2015). Choosing a Health Insurance Plan: Complexity and Consequences. JAMA..

[5] Adepoju O, Mask A, McLeod A (2018). Health Insurance Literacy as a Determinant of Population Health. Popul Health Manag..

[6] Schmid CPR, Beck K, Kauer L. Health Plan Payment in Switzerland. In: Risk Adjustment, Risk Sharing and Premium Regulation in Health Insurance Markets [Internet]. Elsevier; 2018 [cited 2021 Jun 12]. p. 453–89. Available from: https://linkinghub.elsevier.com/retrieve/pii/B9780128113257000166.

[7] Quincy L. Measuring Health Insurance Literacy: A Call to Action, A Report dom the Health Insurance Literacy Expert Roundtable [Internet]. American Institutes for Research. 2012 [cited 2021 Dec 22]. Available from: https://www.air.org/project/measuring-health-insurance-literacy.

[8] Loewenstein G, Friedman JY, McGill B, Ahmad S, Linck S, Sinkula S (2013). Consumers’ misunderstanding of health insurance. J Health Econ..

[9] Wilson CM, Price CW (2010). Do consumers switch to the best supplier?. Oxf Econ Pap..

[10] McCORMACK L, Bann C, Uhrig J, Berkman N, Rudd R (2009). Health Insurance Literacy of Older Adults. J Consum Aff..

[11] Paez KA, Mallery CJ, Noel H, Pugliese C, McSorley VE, Lucado JL (2014). Development of the Health Insurance Literacy Measure (HILM): conceptualizing and measuring consumer ability to choose and use private health insurance. J Health Commun..

[12] Adepoju O, Mask A, McLeod A (2019). Factors Associated With Health Insurance Literacy: Proficiency in Finding, Selecting, and Making Appropriate Decisions. J Healthc Manag.

[13] Paez KA, Mallery CJ. A little knowledge is a risky thing: Wide gap in what people think they know about health insurance and what they actually know. Am Inst Res Issue Brief. 2014;1.

[14] Edward J, Wiggins A, Young MH, Rayens MK (2019). Significant Disparities Exist in Consumer Health Insurance Literacy: Implications for Health Care Reform. HLRP Health Lit Res Pract..

[15] AC Quiroga Gutiérrez Health insurance literacy assessment tools: a systematic literature review. J Public Health. 2021. cited 2021 Sep 16.10.1007/s10389-021-01634-7.

[16] CHPE health survey Wave 1. Center for Health, Policy and Economics, University of Lucerne. 2015

[17] Federal Statistical Office. Schweizerische Gesundheitsbefragung [Internet]. Federal Administration. 2012 [cited 2021 Dec 22]. Available from: https://www.bfs.admin.ch/bfs/de/home/statistiken/gesundheit/erhebungen/sgb.html.

[18] OECD Health policy review. Health Policy in Switzerland [Internet]. 2017 [cited 2021 Dec 21]. Available from: www.oecd.org/health.

[19] OECD. Health at a Glance 2021: OECD Indicators [Internet]. OECD; 2021 [cited 2021 Dec 21]. (Health at a Glance). Available from: https://www.oecd-ilibrary.org/social-issues-migration-health/health-at-a-glance-2021_ae3016b9-en.

[20] Federal Statistical Office. Languages [Internet]. Federal Administration. [cited 2021 Dec 21]. Available from: https://www.bfs.admin.ch/bfs/en/home/statistiken/bevoelkerung/sprachen-religionen/sprachen.html.

[21] R Core Team [Internet]. Vienna, Austria: R Foundation for Statistical Computing; Available from: https://www.R-project.org/.

[22] Epstein J, Santo RM, Guillemin F (2015). A review of guidelines for cross-cultural adaptation of questionnaires could not bring out a consensus. J Clin Epidemiol..

[23] Cronbach LJ (1951). Coefficient alpha and the internal structure of tests. Psychometrika..

[24] Cronbach LJ, Meehl PE (1955). Construct validity in psychological tests. Psychol Bull..

[25] Yasir ASM. Cross Cultural Adaptation & Psychometric Validation of Instruments: Step-wise Description. Int J Psychiatry [Internet]. 2016 Jul 9 [cited 2021 Dec 21];1(1). Available from: https://www.opastonline.com/wp-content/uploads/2016/07/cross-cultural-adaptation-psychometric-validation-of-instruments-step-wise-description-ijp-16-001.pdf.

[26] Portney LG, Watkins MP (2009). Foundations of clinical research: applications to practice.

[27] Fabrigar LR, Wegener DT, MacCallum RC, Strahan EJ (1999). Evaluating the use of exploratory factor analysis in psychological research. Psychol Methods..

[28] Swisher LL, Beckstead JW, Bebeau MJ (2004). Factor Analysis as a Tool for Survey Analysis Using a Professional Role Orientation Inventory as an Example. Phys Ther..

[29] Russell DW (2002). In Search of Underlying Dimensions: The Use (and Abuse) of Factor Analysis in Personality and Social Psychology Bulletin. Pers Soc Psychol Bull..

[30] Browne MW, Cudeck R. Alternative Ways of Assessing Model Fit [Internet]. [cited 2021 Dec 21]. Available from: https://journals.sagepub.com/doi/10.1177/0049124192021002005.

[31] Tran TV. Developing Cross Cultural Measurement [Internet]. New York: Oxford University Press; 2009 [cited 2021 Dec 21]. 160 p. (Pocket Guides to Social Work Research Methods). Available from: https://oxford.universitypressscholarship.com/10.1093/acprof:oso/9780195325089.001.0001/acprof-9780195325089.

[32] Rhudy JL, Arnau RC, Huber FA, Lannon EW, Kuhn BL, Palit S (2020). Examining Configural, Metric, and Scalar Invariance of the Pain Catastrophizing Scale in Native American and Non-Hispanic White Adults in the Oklahoma Study of Native American Pain Risk (OK-SNAP). J Pain Res..

[33] Chen FF (2007). Sensitivity of Goodness of Fit Indexes to Lack of Measurement Invariance. Struct Equ Model Multidiscip J..

[34] Kenny DA, McCoach DB (2003). Effect of the Number of Variables on Measures of Fit in Structural Equation Modeling. Struct Equ Model Multidiscip J..

[35] Federal Statistical Office. Niveau de formation [Internet]. Federal Administration. [cited 2021 Dec 21]. Available from: https://www.bfs.admin.ch/bfs/fr/home/statistiken/bildung-wissenschaft/bildungsstand.html.

[36] Kaiser HF (1974). An index of factorial simplicity. Psychometrika..

[37] Guttman L (1954). A new approach to factor analysis: the Radex. In: Mathematical thinking in the social sciences.

[38] Ruscio J, Roche B (2012). Determining the number of factors to retain in an exploratory factor analysis using comparison data of known factorial structure. Psychol Assess..

[39] Tabachnick BG, Fidell LS (2007). Using multivariate statistics.

[40] Comrey AL, Lee HB. A first course in factor analysis. 2nd ed. London: Psychology Press; 2013. Available from: 10.4324/9781315827506.

[41] Beavers AS, Lounsbury JW, Richards JK, Huck SW, Skolits GJ, Esquivel SL. Practical Considerations for Using Exploratory Factor Analysis in Educational Research. 2013 [cited 2021 Dec 21]; Available from: https://scholarworks.umass.edu/pare/vol18/iss1/6/.

[42] Abdi H, Williams LJ (2010). Tukey’s honestly significant difference (HSD) test. Encyclopedia Res Des.

[43] Abdelmoula M, Chakroun W, Akrout F (2015). THE EFFECT OF SAMPLE SIZE AND THE NUMBER OF ITEMS ON RELIABILITY COEFFICIENTS: ALPHA AND RHÔ: A META-ANALYSIS. Int J Numer Methods Appl..

[44] Shrestha N (2021). Factor Analysis as a Tool for Survey Analysis. Am J Appl Math Stat..

[45] Rhudy JL, Arnau RC, Huber FA, Lannon EW, Kuhn BL, Palit S (2020). Examining Configural, Metric, and Scalar Invariance of the Pain Catastrophizing Scale in Native American and Non-Hispanic White Adults in the Oklahoma Study of Native American Pain Risk (OK-SNAP). J Pain Res..

[46] Peters E, Klein W, Kaufman A, Meilleur L, Dixon A. More Is Not Always Better: Intuitions About Effective Public Policy Can Lead to Unintended Consequences. Soc Issues Policy Rev. 2013 Jan 1;7(1):10.1111/j.1751-2409.2012.01045.x. 10.1111/j.1751-2409.2012.01045.x PMC3758756 24000291

[47] Zhao J, Mir N, Ackermann N, Kaphingst KA, Politi MC (2018). Dissemination of a Web-Based Tool for Supporting Health Insurance Plan Decisions (Show Me Health Plans): Cross-Sectional Observational Study. J Med Internet Res..

[48] Politi MC, Kuzemchak MD, Liu J, Barker AR, Peters E, Ubel PA (2016). Show Me My Health Plans: Using a Decision Aid to Improve Decisions in the Federal Health Insurance Marketplace. MDM Policy Pract..

[49] Brown V, Russell M, Ginter A, Braun B, Little L, Pippidis M (2016). Smart Choice Health Insurance©: A New, Interdisciplinary Program to Enhance Health Insurance Literacy. Health Promot Pract..

[50] Bartholomae S, Russell MB, Braun B, McCoy T (2016). Building Health Insurance Literacy: Evidence from the Smart Choice Health Insurance^TM^ Program. J Fam Econ Issues..

[51] Barnes A, Hanoch Y, Rice T (2014). Determinants of Coverage Decisions in Health Insurance Marketplaces: Consumers’ Decision-Making Abilities and the Amount of Information in Their Choice Environment. Health Serv Res..

