# Supplementary material for: The Swiss Health Insurance Literacy Measure (HILM-CH): Measurement Properties and Cross-Cultural Validation
Source: BMC Health Serv Res. 2023 Jan 26;23:85. doi: 10.1186/s12913-022-08986-0 (PMC9876756; doi:10.1186/s12913-022-08986-0)
Supplement: Supplementary file 1 — Additional file 1. [file 12913_2022_8986_MOESM1_ESM.docx]

Additional file 1: English version of the HILM-CH

Scale 1: confidence in choosing a health insurance plan

|  | Not confident at all  (1) | A little confident  (2) | Reasonably confident  (3) | Quite confident (4) |
| --- | --- | --- | --- | --- |
| … understand the concepts and terms about health insurances? | **** | **** | **** | **** |
| … can estimate what you will have to pay for your care in the coming year (without emergencies)? | **** | **** | **** | **** |
| … know which questions to ask in order to choose the health insurance that is right for you? | **** | **** | **** | **** |
| … know where to find the information you need to choose a health insurance? | **** | **** | **** | **** |
| … know where to go for financial help if you cannot pay your health insurance? | **** | **** | **** | **** |
| … are choosing a health insurance that suits you? | **** | **** | **** | **** |

How confident are you that you…

Scale 2: Comparing health insurance plans

|  | Not likely at all  (1) | A little likely (2) | Reasonably likely  (3) | Very likely (4) |
| --- | --- | --- | --- | --- |
| … can find out if an insurance policy covers unexpected costs, such as hospitalization? | **** | **** | **** | **** |
| … understand what you would have to pay for a visit to the emergency room? | **** | **** | **** | **** |
| … can find out how much you have to pay yourself for a visit to a medical specialist? | **** | **** | **** | **** |
| … can find out how much you have to pay for medicine on prescription? | **** | **** | **** | **** |
| … can find out which doctors and hospitals are covered by an insurance policy? | **** | **** | **** | **** |
| … can find out what the differences are between insurance policies? | **** | **** | **** | **** |
| … can find out if you have to pay for certain care yourself? | **** | **** | **** | **** |

When comparing health insurance plans how likely is it that you…

Scale 3: Confidence in using a health insurance plan

|  | Not confident at all  (1) | A little confident  (2) | Reasonably confident  (3) | Quite confident (4) |
| --- | --- | --- | --- | --- |
| … know what to do if your health insurance company refuses to pay for care that you think you should be reimbursed? | **** | **** | **** | **** |
| … can find out how much you have to pay out of your own pocket? | **** | **** | **** | **** |
| … can find out how much of the costs your health insurance will reimburse? | **** | **** | **** | **** |
| … know which questions to ask your health insurance company if you have a problem with a reimbursement? | **** | **** | **** | **** |

Before you receive certain care, how confident are you that you…

Scale 4: Proactive use of the health insurance plan

|  | Not likely at all  (1) | A little likely (2) | Reasonably likely  (3) | Very likely (4) |
| --- | --- | --- | --- | --- |
| … will find out what is and is not covered by your health insurance before you receive certain care? | **** | **** | **** | **** |
| … will contact customer service to ask what care is covered by your health insurance? | **** | **** | **** | **** |
| … can find out whether a doctor has a contract with your health insurer before you visit that doctor? | **** | **** | **** | **** |
| … look at the overviews of your health insurance to see what you still have to pay and what the health insurance company has reimbursed? | **** | **** | **** | **** |

How likely is it that you…
