# Supplementary material for: The Swiss Health Insurance Literacy Measure (HILM-CH): Measurement Properties and Cross-Cultural Validation
Source: BMC Health Serv Res. 2023 Jan 26;23:85. doi: 10.1186/s12913-022-08986-0 (PMC9876756; doi:10.1186/s12913-022-08986-0)
Supplement: Supplementary file 2 — Additional file 2. [file 12913_2022_8986_MOESM2_ESM.docx]

Additional Table 1. Basic description of the HILM-CH instrument

| Items | Mean | SD | Skewness | | Kurtosis | | | Floor (%) | | Ceiling (%) | |
| --- | --- | --- | --- | --- | --- | --- | --- | --- | --- | --- | --- |
| Scale 1: confidence in choosing | | | | | | | | | | | |
| 1 | 2.7966 | 0.7237 | -0.2860 | | 2.957 | | 3.88 | | | 14.07 | |
| 2 | 2.8776 | 0.7499 | -0.4108 | | 3.0364 | | 4.41 | | | 18.06 | |
| 3 | 2.8988 | 0.7296 | -0.3498 | | 2.9976 | | 3.28 | | | 18.84 | |
| 4 | 3.0389 | 0.7232 | -0.4344 | | 3.0329 | | 2.37 | | | 25.35 | |
| 5 | 2.5704 | 0.9610 | -0.1290 | | 2.0676 | | 15.77 | | | 18.01 | |
| 6 | 2.9961 | 0.7072 | -0.3544 | | 3.0097 | | 2.05 | | | 22.45 | |
| Scale 2: comparing health plans | | | | | | | | | | | |
| 7 | 2.9278 | 0.7521 | -0.3045 | | 2.7385 | | | 3.03 | | 21.67 | |
| 8 | 2.6481 | 0.8259 | -0.0699 | | 2.4303 | | | 7.37 | | 15.28 | |
| 9 | 2.7706 | 0.8148 | -0.1825 | | 2.4736 | | | 5.63 | | 18.77 | |
| 10 | 2.8037 | 0.8112 | -0.2035 | | 2.4767 | | | 5.14 | | 19.88 | |
| 11 | 2.9468 | 0.7747 | -0.3327 | | 2.6506 | | | 3.35 | | 23.59 | |
| 12 | 2.7991 | 0.7662 | -0.2030 | | 2.6548 | | | 4.17 | | 17.00 | |
| 13 | 2.8384 | 0.7746 | -0.2410 | | 2.6389 | | | 4.16 | | 18.92 | |
| Scale 3: confidence using | | | | | | | | | | | |
| 14 | 2.4393 | 0.8863 | | .01022 | | 2.2855 | | | 14.89 | | 11.99 |
| 15 | 2.8004 | 0.7756 | | -0.2056 | | 2.6172 | | | 4.37 | | 17.53 |
| 16 | 2.8201 | 0.7698 | | -0.2284 | | 2.6590 | | | 4.13 | | 17.81 |
| 17 | 2.9302 | 0.7959 | | -0.3361 | | 2.684 | | | 3.63 | | 24.37 |
| Scale 4: being proactive | | | | | | | | | | | |
| 18 | 2.9380 | 0.7938 | | -0.3361 | | 2.5813 | | | 3.63 | | 24.37 |
| 19 | 3.1720 | 0.7760 | | -0.5808 | | 2.6712 | | | 2.14 | | 37.29 |
| 20 | 2.8261 | 0.9189 | | -0.2879 | | 2.1821 | | | 8.43 | | 26.18 |
| 21 | 3.0883 | 0.8109 | | -0.5690 | | 2.7188 | | | 3.53 | | 34.51 |
| Source: Swiss Health Insurance Literacy Survey 2021. | | | | | | | | | | | |

Additional Table 1 shows the results of the descriptive analysis for each of the scores of the HILM-CH items according to the EFA model. The mean and standard deviation of the item score indicated that respondents tend to have positive self-perceived confidence and behavior. Floor and ceiling effects are present in the sample if more than 15% of the respondents answered the lowest or highest score (1). In this analysis, floor effects could be detected for none of the items. However, except for items 1 and 14, all items had ceiling effects. Skewness and Kurtosis ranged from -0.57 to 0.09 and 2.08 to 3.03. Three items had kurtosis coefficients larger than 3.0, depicting data distributed with shape peaks and long and flat tails (2).

1. Terwee CB, Bot SDM, de Boer MR, van der Windt DAWM, Knol DL, Dekker J, et al. Quality criteria were proposed for measurement properties of health status questionnaires. J Clin Epidemiol. 2007 Jan;60(1):34–42.

2. Westfall PH. Kurtosis as Peakedness, 1905 – 2014. R.I.P. Am Stat. 2014;68(3):191–5.
