# Supplementary material for: The Swiss Health Insurance Literacy Measure (HILM-CH): Measurement Properties and Cross-Cultural Validation
Source: BMC Health Serv Res. 2023 Jan 26;23:85. doi: 10.1186/s12913-022-08986-0 (PMC9876756; doi:10.1186/s12913-022-08986-0)
Supplement: Supplementary file 3 — Additional file 3. [file 12913_2022_8986_MOESM3_ESM.docx]

Additional Table 2. ANOVA post hoc weighted pairwise comparisons using Tukey’s honestly significant difference (HSD) method

|  | **French vs. German** | | **Italian vs. German** | | **Italian vs. French** | |
| --- | --- | --- | --- | --- | --- | --- |
| **VARIABLES** | **Mean difference (SD)** | **Adjusted p-value** | **Mean difference (SD)** | **Adjusted p-value** | **Mean difference (SD)** | **Adjusted p-value** |
| Age | -1.209 (0.421) | 0.01 | -3.11 (0.76) | <0.001 | -1.90 (0.82) | 0.05 |
| Gender | 0.00 (0.02) | 0.96 | -0.06 (0.03) | 0.11 | -0.06 (0.03) | 0.11 |
| Nationality | 0.04 (0.01) | <0.001 | 0.22 (0.02) | <0.001 | 0.18 (0.02) | <0.001 |
| Education | 0.07 (0.01) | <0.001 | 0.06 (0.03) | 0.06 | -0.01 (0.03) | 0.98 |
| Chronic condition | 0.02 (0.02) | 0.62 | 0.11 (0.03) | <0.001 | 0.10 (0.03) | <0.001 |
| Number of doctor visits | -0.14 (0.21) | 0.78 | -0.33 (0.39) | 0.67 | -0.19 (0.42) | 0.89 |
| Deductible level | -0.12 (0.07) | 0.18 | 0.16 (0.13) | 0.40 | 0.29 (0.14) | 0.09 |
| Model type | 0.15 (0.04) | <0.001 | 0.16 (0.08) | 0.09 | 0.01 (0.08) | 0.99 |
| Out-of-pocket expenditures | 0.05 (0.06) | 0.71 | -0.09 (0.10) | 0.63 | -0.14 (0.11) | 0.42 |
| Source: Swiss Health Insurance Literacy Survey 2021. | | | | | | |

Additional Table 2 reports the results of post hoc tests after ANOVA. While ANOVA allows for tests for differences across groups, it does not identify which differences between pairs are significant. Because Switzerland is a multicultural country, we expect some significant differences across the linguistic groups (German, French, and Italian). Therefore, Tukey’s honestly significant difference (HSD) method was used to compare all possible group pairings (1). Adjusted p-values are reported to identify the group’s significantly different comparisons.

The table confirms some significant cultural differences between Switzerland's German- and Latin-speaking parts. There are no significant differences between the groups regarding gender, number of doctor visits in the last 12 months, deductible level, and out-of-pocket expenditures. On the contrary, there are some statistical differences for the Swiss nationality variable. Finally, we notice that Italian speakers are younger than German speakers. Swiss Germans tend to have more years of education than French speakers, and Italian speakers have more chronic conditions than the rest of the population.

1. Abdi H, Williams LJ. Tukey’s Honestly Signiﬁcant Diﬀerence (HSD) Test. :5.
