# Supplementary material for: The Swiss Health Insurance Literacy Measure (HILM-CH): Measurement Properties and Cross-Cultural Validation
Source: BMC Health Serv Res. 2023 Jan 26;23:85. doi: 10.1186/s12913-022-08986-0 (PMC9876756; doi:10.1186/s12913-022-08986-0)
Supplement: Supplementary file 4 — Additional file 4. [file 12913_2022_8986_MOESM4_ESM.docx]

Additional file 2. Correlations of the 4-factor model factors

This figure shows the bivariate distribution of the four factors based on their predicted values. Scatter plots indicate the weighted bivariate distribution of the four factors. They allow seeing that individuals who scored high on a certain scale also score high on another scale. Regression line and rho correlation coefficients are included (in blue and red, respectively). Margin histograms show each factor's distribution, with the mean depicted by a blue dashed line.
